# Picturing aesthetic futures: values and visual tools within shared decision-making

**DOI:** 10.1136/ihj-2022-000126

**Published:** 2022-09-12

**Authors:** Graham Pullin, Alan Cribb

**Affiliations:** 1 DJCAD, University of Dundee, Dundee, UK; 2 King's College London, London, UK

**Keywords:** healthcare quality improvement, patient-centred care, patient preference, rehabilitation, shared decision making

In person-centred healthcare improvement, design thinking^1^ is being applied to multidisciplinary healthcare practices. In this paper, we will argue for design methods to operate within, and to support the integration of, everyday healthcare interactions. We draw on two specific public participation studies related to deepening patients' agency in prosthetic services, using visual tools to explore aesthetic decisions, but we suggest these have much broader relevance.

## Shared decision-making, values and tools

Shared decision-making, it is generally accepted, involves eliciting people’s values.^2^ It is not just about negotiation around information^3^ but, ideally, about trying to ensure that decisions reflect what really matters to people. However, incorporating values is not easy.

Consultation can be framed as professionals communicating ‘advantages and disadvantages’ and providing ‘evidence-based treatment information’.^4^ Yet there are areas where values are so contested that it becomes extremely difficult to separate ‘advantages’ from ‘disadvantages’, sometimes even challenging the goals of healthcare. Many disabled people, for instance, may feel at odds with the way that disability can be framed in healthcare.^5^ Under these circumstances it is arguably even more important and challenging to elicit values.

‘Shared decision-making tools’ or ‘patient decision aids’^6^ can play a role in preparing and empowering patients to think through what is important to them. Currently, these tools are predominantly text-based, although some include visual representations of statistical outcomes.^7^

We offer a complementary example where the role of visualisation is rather different. In decisions relating to aesthetics, visual methods can help represent the *substance* of a possible choice, not just the relevant facts. Rather than helping to weigh up alternatives from a more objective distance, they can provide an opportunity to be immersed in subjective possibilities.

## A disconnection between values and aesthetics in the choice of a prosthesis

In choices of prosthetics, for example, values underpin aesthetics. A cosmetic imitation of a human hand has, however unintentionally, connotations of fixing a body that is inadequate. This might suggest that its wearer is attempting to conceal their disability: to ‘pass’ as nondisabled—implying a stigma which may be anathema to many people with limb difference. These are just part of the meanings of prosthetics in the contexts of bodies and lives.^8^

So we might expect just such deeply personal—even political—issues to be an important foundation of consultations in limb fitting services,^9^ with prosthetists and psychologists. Yet when we have talked with wearers this does not seem to be the case. One reflected that the aesthetics of her prostheses were an afterthought: ‘there was no discussion into it’. Another observed that her prosthetist had fixed views on the (gendered) choices that male and female amputees should make and far from welcoming a discussion of values, seemed to resent this as a challenge to her professional judgement.

A limited perspective is also evident in patient reported outcome measures (PROMs). The Prosthetics Evaluation Questionnaire (PEQ)^10^ includes careful assessment of the physical comfort of a prosthesis, yet not of how comfortable its wearer feels about whether it represents their identity. Current measures view aesthetics through an impoverished lens:

‘Over the past four weeks, rate how your prosthesis has looked’ on a scale from TERRIBLE to EXCELLENT. (PEQ: Q.3J)^10^

The alternative Trinity Amputation and Prosthesis Experience Scales-Revised (TAPES-R) PROM informed though it is by an approach of ‘psychoprosthetics’,^11^ which augments a biomedical perspective with a psychosocial lens, does little more in respect to aesthetics. PROMs have a valuable role in healthcare improvement but values that they fail to capture are likely to remain overlooked and underdiscussed. Part of the challenge here is that issues may not easily translate into words.^12^ Here is one highly articulate person with limb difference describing what their prosthetic hand is, to them:

It’s difficult to describe. It’s sort of like shoes or glasses or… it’s part of you but it’s not a *hand*: it’s a hand, if that makes sense…^13^

New visual tools can, we believe, support more open and nuanced communication between clinicians and patients.

## An engaging and integrating visual tool: ‘Hand of you’

One such visual tool was deployed in the context of a public exhibition at the V&A Museum of Design, Dundee.^13^ An estimated 140 000 visitors to the museum—disabled and non-disabled, healthcare professionals and potential patients—were invited to choose a prosthetic hand, and to relate this choice to their own values. The aim of this exhibition was to catalyse public reflection and discussion about the potential roles both of disability in design and of design within healthcare. It combined public engagement with qualitative research.

No personal data were collected; all responses were anonymous although participants were invited to identify as ‘a person with limb difference’, ‘a disabled person’ or ‘a healthcare professional’. Responses from people under 18 were removed before analysis.

The exercise was framed by a single sheet of A3 paper, preprinted yet incomplete; constrained in some ways and open-ended in others. Participants were offered a choice of four photographs of different prosthetic hands: a ‘cosmetic’ life-like silicone glove; an overtly robotic-looking hand; a hand worn inside a black rubber glove and—importantly—a blank sheet representing not wearing a prosthesis, acknowledging this as a potential positive choice (rather than ‘non-compliance’).

It should be noted that the questions were not all directly about a choice of prosthesis: they included an invitation to 'introduce yourself' by choosing a cuff of a garment and reflecting on where you might wear this and what it would say to the world about who you are. This tangential approach, demanding an imaginative, creative as well as thoughtful response, is well established in design research methods.^14^

**Figure 1:**
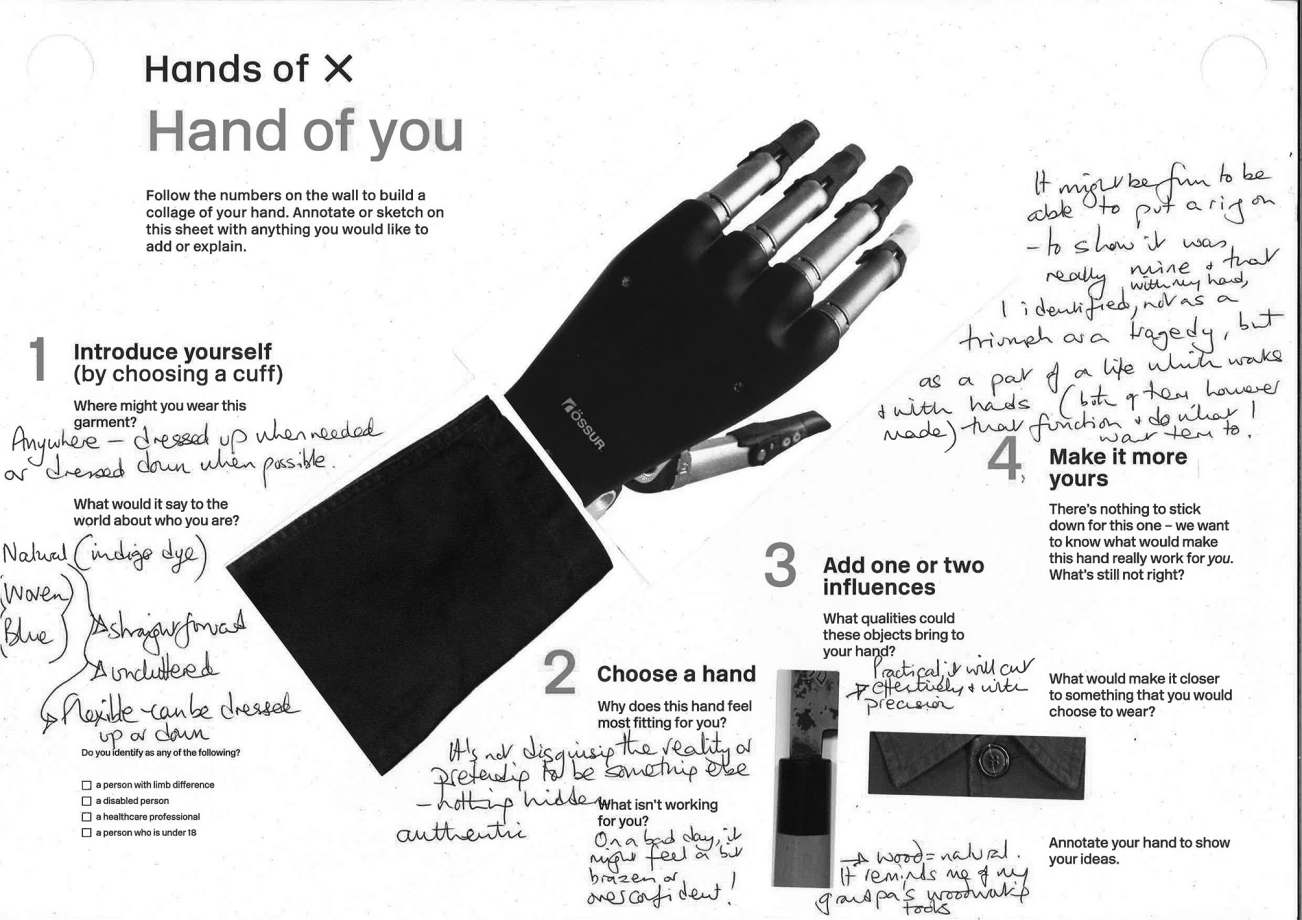
An example of a ‘Hand of you’ sheet, 1 of 7000, showing collage and annotation. We confirm that we have permission to reuse this image.

Another set of photographs consisted of abstract visual details of non-medical objects—glasses; clothing; utensils—that might relate to perceptions of a personal object. Participants were able to add these and, importantly, were asked to explain their reasons (figure 1).

The results evidence that this was widely accessible and engaging. Over 7000 visitors completed the exercise which, taking 5–10 min, was not an insignificant demand in a museum, yet a perfectly feasible investment of time within a healthcare practice.

The completed sheets were pinned up within the exhibition and served as a visualisation of the diverse and contradictory responses of such a broad cross-section of the public and healthcare sector, underlining why generalised assumptions are inappropriate and shared decision-making is vital.

**Figure 2:**
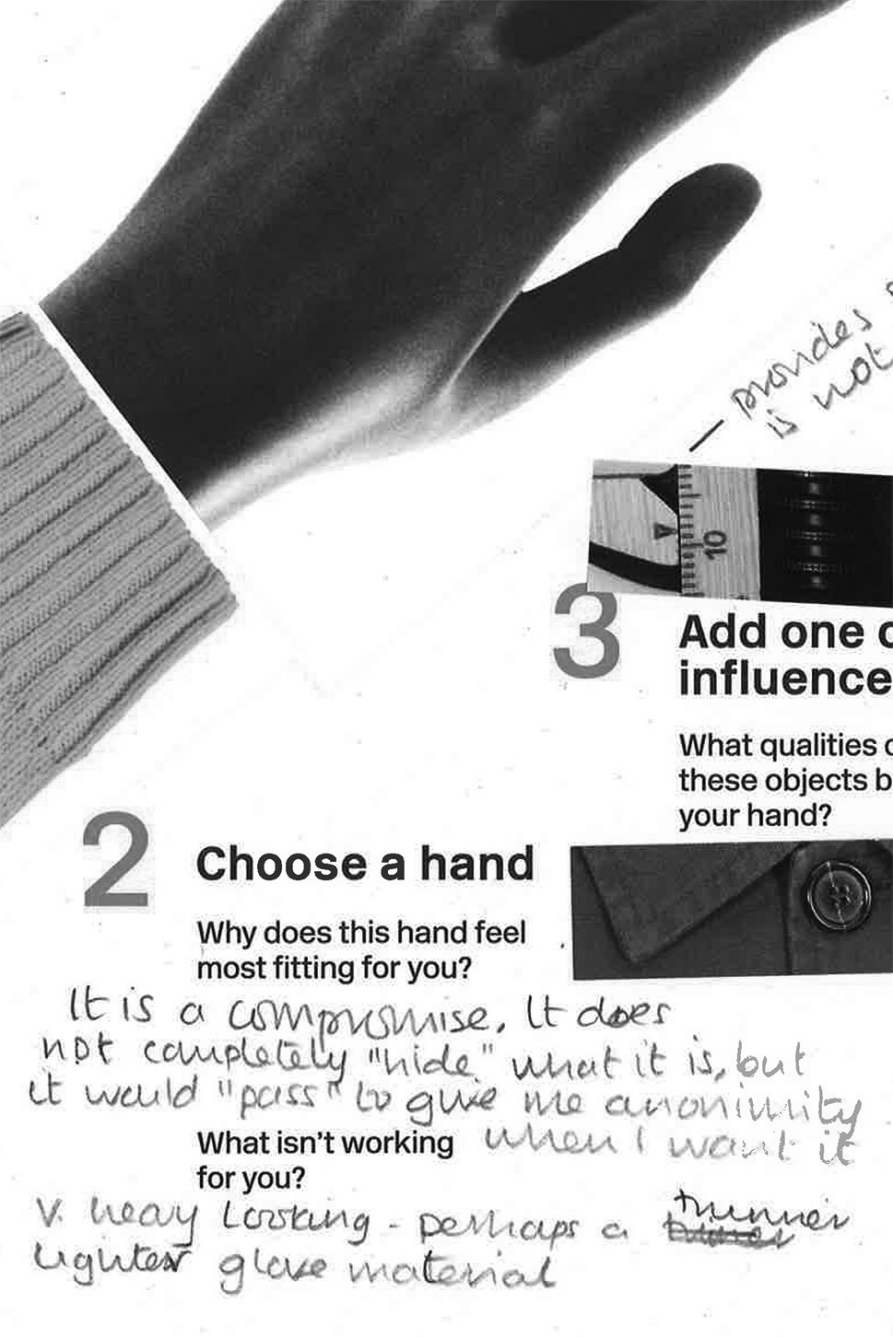
A detail of a second ‘Hand of you’ sheet, eliciting reflection on a knowing compromise. We confirm that we have permission to reuse this image.

The responses illustrated not only preferences, but the dilemmas and values behind these choices (figure 2). Examples included several about managing identity as a disabled person:

I feel a tension between wanting to make it look as 'normal hand' as possible vs. embracing technology and opportunity.

It doesn't try to hide or mask what has happened. It shows that I have accepted this & moved on.^13^

## Prototyping and codesign within shared decision-making

The collaging exercise is a form of prototyping—modelling future possibilities to enable reflection. Prototyping is an art not a science and is always a balance between representing the eventual (potential) outcome, and making it clear that this is not exact in every detail. The images of prostheses were printed as grainy black and white halftones so that in the case of the cosmetic glove, the skin colour and ethnicity implied was open to interpretation. Even the perspective was deliberately askew to undermine any handedness of the images and to add an informality. The visual language set the spirit in which people engaged, which is why skilled graphic designers could play a larger role in visual decision-making tools.

While it is obvious that codesign inherently involves shared decision making,^15^ the reciprocal relationship is arguably less prominent. Such visual tools would incorporate codesign into shared decision-making.

On a previous research project, Hands of X, visual tools were deployed to support decision-making in the context of a speculative healthcare service. A method of experience prototyping was used, in which the act of choosing was prototyped, without this involving functioning prototype prostheses or clinical trials. The service was realised as an installation in a fashionable eyewear retailer: this context influencing the frame of mind in which the participants engaged with it. In-depth qualitative data were elicited by encouraging participant reflections and insights. The sample consisted of four prosthetic wearers each of whom contributed rich material over an extended period (a total participation of around 3–4 hours each).

Wearers could more fully codesign hands in combinations of materials, specifying a hand that they felt represented them. They were able to assemble prototypes from jigsaw pieces of the actual materials: woods, acetates and leathers. After trying out combinations, recombining and comparing, almost all of our testers arrived at a point where they said something like ‘that’s it!’, or even ‘that’s my hand’.^8^

One wearer reflected on his choice: ‘I like this wood. I like its colour. I like the neutrality of it. It’s warm as well and there’s an unthreateningness about wood that certainly I feel is much more fitting to who I think I am and what I would like to wear and the perception that I would like to give out to people’.^8^

## Decisions and relationships over time

Parsons characterises an important decision as ‘a choice that reflects the kinds of values that help shape our lives’, ‘a ‘ritual process’ by which we give meaning and structure to the world’.^16^ Choice is not only informed by pre-existing values but can be the process through which values are crystallised. Shared decision-making can sometimes be about enabling people to ‘create’ their identities and future lives.

A patient’s relationship with their prosthetist can last 20 years or more,^17^ so there are normally opportunities to revisit decisions. What is the most appropriate prosthesis may evolve in the light of a patient’s lived experience. Use can inflect and inform aesthetic responses, deepening our relationships with objects over time, whereas this richer temporal perspective is typically missing from healthcare language. The assessment in PROMs of appearance ‘over the last 4 weeks’ omits a great deal. This could preclude the possibility of an *increased* sense of ownership and appreciation—an insight that emerged, unprompted, in some of the ‘Hand of you’ responses:

I take inspiration from my wok, gets better with age.

It reminds me of my grandpa’s woodworking tools.^13^

## Aesthetics and integrated healthcare

The intersection of disability and aesthetics introduces complexities that are virtually impossible to discuss through text and numbers alone. We argue that carefully designed and creatively engaging visual tools can play a crucial role here. We would also suggest that these insights have broader relevance for extending and strengthening shared decision-making practices. There is scope for adopting and adapting a broad prototyping approach for many kinds of healthcare decisions—encouraging and helping people to imagine future lives with or without various treatments, objects, adaptations to environments and so on. It is accepted that ‘future research is needed to develop novel decision aids for other […] key decisions’^6^ including more ‘communicative aspects’.^4^ While the International Patient Decision Aids Standards helpfully contribute to evidence-based practice, at the same time, we need to ensure that they do not stifle either more critical thought^18^ or more creative explorations.

Although arts-based practices—creative workshops, art therapy, dance activity—are not uncommon in healthcare, they are usually complementary activities, separate from core clinical practices. Whereas we are stressing that design practices could sometimes form part of the core interaction between clinicians and patients, helping to subtly reframe and deepen these relationships so as to enrich healthcare.^19^

More broadly we can see a role for a forum to consider aesthetic issues that currently are overlooked by both medical science and medical ethics. We could, for example, envisage a network bringing together healthcare professionals and service providers, disabled people, designers and bioethicists, supporting a joined-up while value-based approach^20^; a Forum for Healthcare Aesthetics.
